# Biosynthesis pathways of expanding carbon chains for producing advanced biofuels

**DOI:** 10.1186/s13068-023-02340-0

**Published:** 2023-07-04

**Authors:** Haifeng Su, JiaFu Lin

**Affiliations:** 1 Key Laboratory of Degraded and Unused Land Consolidation Engineering, The Ministry of Natural and Resources, Xian, 710075 Shanxi China; 2 Antibiotics Research and Re-Evaluation Key Laboratory of Sichuan Province, Sichuan Industrial Institute of Antibiotics, School of Pharmacy, Chengdu University, Chengdu, 610106 China

**Keywords:** Carbon chains, Expanding, Advanced biofuels, CRISPR/Cas9

## Abstract

Because the thermodynamic property is closer to gasoline, advanced biofuels (C ≥ 6) are appealing for replacing non-renewable fossil fuels using biosynthesis method that has presented a promising approach. Synthesizing advanced biofuels (C ≥ 6), in general, requires the expansion of carbon chains from three carbon atoms to more than six carbon atoms. Despite some specific biosynthesis pathways that have been developed in recent years, adequate summary is still lacking on how to obtain an effective metabolic pathway. Review of biosynthesis pathways for expanding carbon chains will be conducive to selecting, optimizing and discovering novel synthetic route to obtain new advanced biofuels. Herein, we first highlighted challenges on expanding carbon chains, followed by presentation of two biosynthesis strategies and review of three different types of biosynthesis pathways of carbon chain expansion for synthesizing advanced biofuels. Finally, we provided an outlook for the introduction of gene-editing technology in the development of new biosynthesis pathways of carbon chain expansion.

## Introduction

**Fig. 1:**
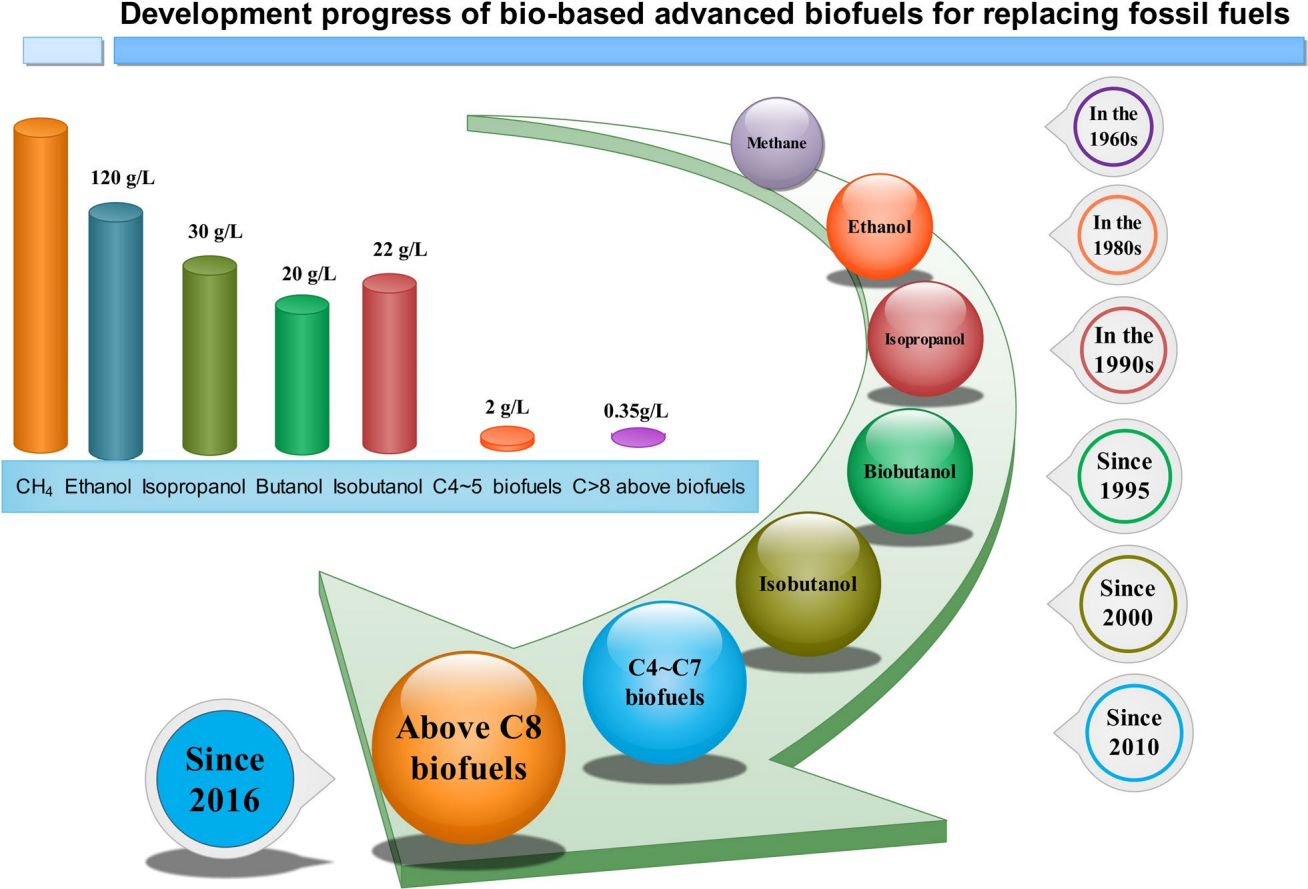
Development progress of bio-based small molecule compounds for replacing petroleum industry. With the development of bio-based small molecule compounds for substituting non-renewable gasoline, increasing length of carbon chain has been a hallmark of biosynthesis strategy. One of the major future developments of bio-based small molecule compounds for replacing fossil fuel is to exploit advanced biofuels with long carbon chains (C > 6) using appropriate engineering strategy

Fossil fuels such as gasoline, have increasingly incited a string of ecological disaster, environmental pollution, and looming international policy issues [1]. Developing advanced biofuels to substitute for petrochemical production through biotransformation is regarded as a promising alternative [2]. The production of renewable biofuels through bioengineering has long been executed, converting single-carbon methane to longer biofuels closer to the octane value (C_8_H_18_) of gasoline (Fig. 1). Due to various remarkable defects related to its chemical properties, such as high hydroscopicity and low energy density, recent research has attempted to substitute some traditional bioenergy compounds, like ethanol and butanol, with other advanced biofuels that process long carbon chain (C ≥ 6 and ≤ 10) such as fatty alcohol compounds, olefins and alkanes compounds. In fact, recent findings have reported important progress in the expansion of carbon chains via using novel engineering strategy to obtain advanced biofuels with long carbon chain [3].

Some biosynthesis pathways have been proposed for synthesizing advanced biofuels with long carbon chains, such as leveraging the 2-ketonic acid metabolic pathway [4] and the fatty acid metabolic pathway [5]. In the last decade, studies on advanced biofuels with long carbon chains have attracted more attention, however, the problems, such as low efficiency, have kept these pathways from being adopted for industrialization. Biosynthesis pathways directly determine the choice of metabolic pathways of carbon chain expansion and further ascertain the specific key enzymes of the target product. Therefore, it is necessary to review existing biosynthesis pathways and construct new metabolic pathways.

In this paper, we first presented the low efficiency problem of expanding carbon chains and then put forth two biosynthesis strategies to overcome the problem. Based on these biosynthesis strategies, three different biosynthesis pathways were thoroughly reviewed according to classification. Furthermore, some methods including CRISPR/Cas9 gene-editing technology were introduced to improve the low efficiency, and to increase the speed of creating new biosynthesis pathways. This review will be conducive to providing valuable lessons for synthesizing advanced biofuels in future.

## Low efficiency of expanding carbon chains

**Fig. 2:**
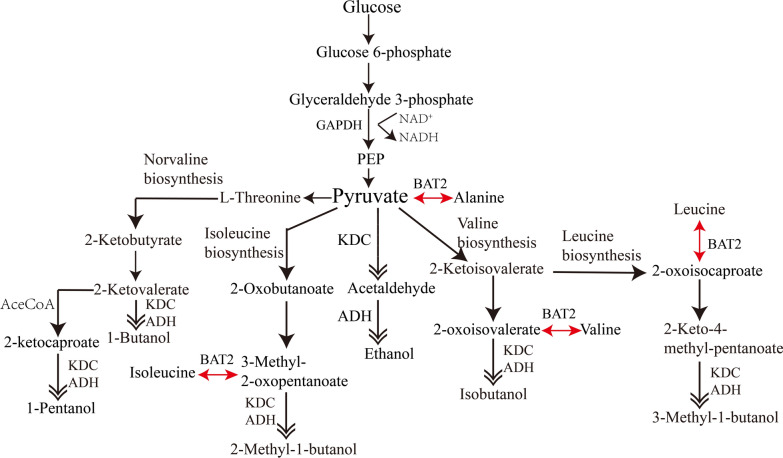
Engineering strategy for producing short linear alcohols (C2–C5) based on the natural characteristics of microorganisms. Generally, synthesizing bio-based short linear alcohols (C2–C5) is based on the Ehrlich pathway and α-keto acid pathway of the microorganism itself. This engineering strategy is only possible to reach a maximum of five carbons, which cannot meet the necessary for extending carbon chain in generating non-natural bio-based small molecule compounds (C > 6) for substituting non-renewable gasoline. GAPDH: glyceraldehyde 3-phosphate dehydrogenase. *PEP* phosphoenolpyruvate. *KDC* 2-keto acid decarboxylases. *ADH* alcohol dehydrogenase. *BAT2* aminotransferase. Double arrow show the oxidation–reduction pathway to biofuel

The production of diversified biofuels with short linear carbon chain (C < 5) is generally derived from a biosynthesis pathway that relies on the 2-keto acid pathway (Fig. 2), an intrinsic metabolic pathway in microorganism such as yeast, without requiring the expansion of carbon chain [6]. However, the number of carbon atoms derived from this approach is limited to a maximum of 5 carbons for linear alcohol except for cyclic alcohols such as 2-phenylethanol [19]. Therefore, it is necessary to develop other biosynthesis pathways that would expand carbon chains (C ≥ 6) to obtain linear advanced biofuels that are closer to gasoline.

The majority of native microorganisms isolated from their natural environment are inefficient in synthesizing advanced biofuels. To achieve production of advanced biofuels, exogenetic metabolic pathways need to be successfully incubated in native microorganisms, which usually requires creating an engineering strategy that can overexpress multi-enzyme systems [7].

The process will inevitably produce low efficiency problem. Firstly, it involves more gene expression systems, more metabolic pathways, and more associated intermediates, which will increase the probability of failure, such as overproduction of unnecessary by-products or a low output of targeted products. Secondly, fine-tuned regulation of microbial physiology in the process of controlling the expression systems is complicated by the import of diversiform multi-enzyme combined system. Finally, technical difficulty in identifying a single engineering event from the entirety of the microbial genome also greatly increases the probability of failure. A good synthesis strategy can solve the above problems to some extent. Therefore, biosynthesis strategies are of great significance to guide effective metabolic pathways of biosynthesis. Only by involving sophisticated metabolic pathways can lay the foundation for solving low efficiency problem. So far, however, although a number of biosynthetic pathways have been developed for producing different advanced biofuels, how to choose a reasonable synthesis strategy is rarely summarized for improving the synthesis efficiency.

## Biosynthesis strategies on advanced biofuels

Generally, obtaining biosynthetic pathways can be divided into three types according to the source: (1) use only the host's own metabolic pathway. For example, the metabolic pathway of *Escherichia coli* itself: the synthesis pathway of metabolites such as succinic acid, pyruvate, l-threonine and l-valine already exists in the natural *E. coli* metabolic pathway [4, 8]. The metabolic pathway of *E. coli* itself should be modified and regulated appropriately, such as releasing feedback inhibition of key sites, improving the expression of rate-limiting enzymes, regulating the metabolic balance using cofactors, etc., to maximize the metabolic flow to target products. (2) Introduction of foreign metabolic pathways: For example, *E. coli* has limited genes, so it is often necessary to use foreign genes to complete the metabolic pathway or improve the efficiency of the original metabolic pathway. For example, glycerol-3-phosphate dehydrogenase, glycerol-3-phosphatase from *S. cerevisiae* and glycerol-3-phosphatase from *Klebsiella pneumoniae* were introduced into the *E. coli* cell factory to produce 1, 3-propylene glycol [9, 10]. An alanine dehydrogenase derived from *Geobacillus stearothermophilus* was introduced to the cell factory to produce L-alanine. Five foreign genes from different sources were integrated into *E. coli* cell factory to produce 1,4-butanediol [11]. (3) Creating biosynthetic pathways that do not exist in nature microorganism. With the help of technologies such as rational protein modification and de novo design, the design of biosynthetic pathways has also broken through the limitations of natural pathways and gradually developed non-natural biosynthetic pathways.

**Fig. 3:**
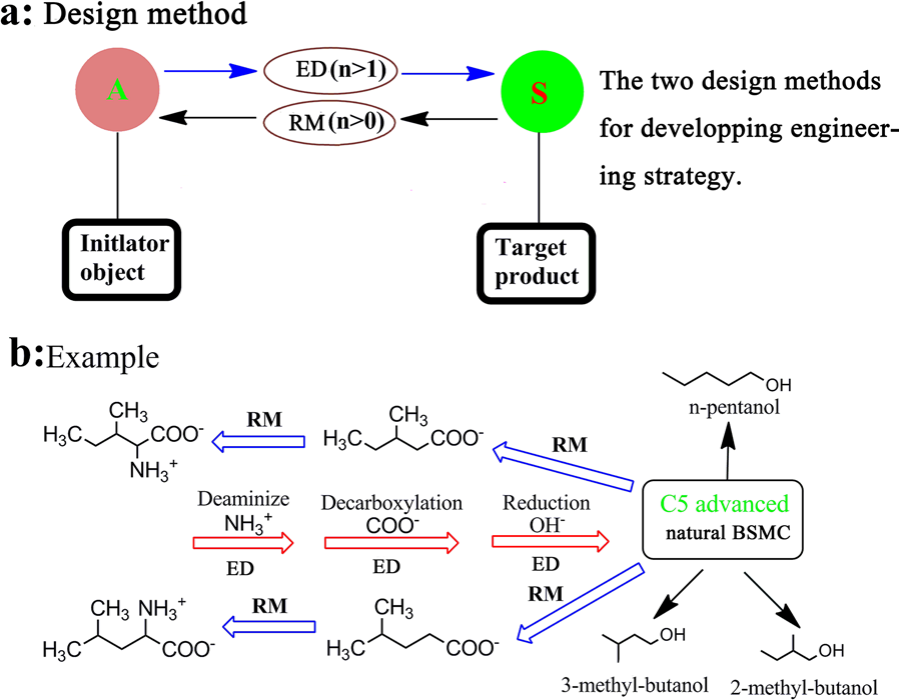
The two biosynthesis strategies of expanding carbon chain in exploring new biosynthesis pathway. *RM* retrospective method. *ED* evolutionary design method. *n* reaction steps

However, biosynthesis strategies play a fundamental role in the initial exploration of novel biosynthetic pathways. Here, we summarized two biosynthesis strategies based on the above three types of biosynthetic pathways. To achieve advanced biofuels and overcome low efficiency problem of expanding carbon chains for the exploration of biosynthetic pathways, there are two basic biosynthesis strategies to be followed in the synthesis of advanced biofuels. As shown in Fig. 3, the first biosynthesis strategy is a retrospective method (RM) based on the molecular structure of the target product, which involves the reverse inference of substrates with similar structures (Fig. 3a). This strategy relies on reverse deduction and de novo analysis of the whole metabolic network of the target product, as well as the optimization of multi-step metabolic pathways. This strategy has great potential for obtaining new metabolic pathways, however, it requires the identification of multitudinous enzymes. For example, based on RM, through reversely deducing and analyzing analogous molecular structure of the target product 1,4-butanediol, a comparative study was conducted via analyzing potential metabolic pathways [12, 13]. An optimal engineering pathway in *E. coli* was designed using only four steps with Acetyl-CoA, α-ketoglutarate, glutamic acid, and succinyl Coenzyme A to achieve a maximum production of 18 g/L of 1,4-butanediol [13].

The second biosynthesis strategy is an evolutionary design (ED) approach based on known metabolic pathways or molecular structures of molecules similar to advanced biofuels (Fig. 3b). This strategy takes full advantage of the abundance of biological information in different databases, such as KEGG, NCBI and protein database, and extrapolates the appropriate extrinsic engineering pathway for synthesizing the target products. This strategy could identify the longest and shortest engineering pathway by tracking the metabolic flux of elements. Once a particular chemical structure similar to the target product among the intermediate metabolites, such as carbon metabolic intermediate was determined, the feasibilities of all potential engineering pathways will be validated according to the metabolic flux of the target product. For example, according to the molecular structure of branched-chain amino acids, such as leucine and isoleucine, the metabolic pathways were recombined to produce advanced biofuels hexanol [14, 15] and 3-methyl-butanol [16, 17]. This strategy provides an inspiration for discovering and designing novel biosynthetic pathways based on known metabolic pathways. In addition to using biological information, some prediction tools of synthetic pathways such as network-based pathway analysis tool could also be employed in this strategy, which could speed up the discovery of new biosynthetic pathways for synthesizing advanced biofuels.

Based on the above two biosynthetic strategies, the optimal metabolic pathway can be determined to solve the problem of low efficiency to a large extent in the process of extending the carbon chain, such as reducing the intermediate metabolic reaction as much as possible, and reducing the metabolic tributaries producing by-products as much as possible. Through the above strategies, obtaining the metabolic pathway with the fewest reaction steps can reduce the difficulty in the technical level of gene manipulation, for example, it is easier to knock out the by-product generation pathway, more conducive to release the inhibition of product synthesis, the stability of key enzymes in the overexpression synthesis pathway, the calculation of the minimum metabolic network model, the assembly of metabolic pathway, and the dynamic regulation of gene expression.

## Biosynthetic pathways of advanced biofuels via expanding carbon chains

To date, different biosynthetic pathways of expanding carbon chains have been developed based on the above strategies using multi-enzyme expression system to acquire advanced biofuels including C6–C10 alcohols, C6–C18 alkanes, enolates, and C6–C10 olefins [18]. Here, we reviewed three important biosynthetic pathways of expanding carbon chains using 2-keto acid intermediates, fatty acid intermediates and reverse β-oxidation intermediates, and performed the analysis using concrete examples.

### Production of advanced biofuels using 2-keto acid intermediates

**Fig. 4:**
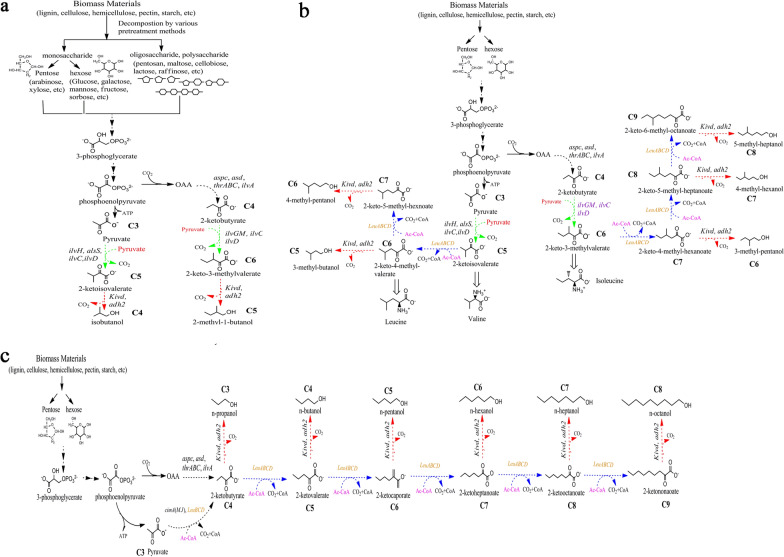
Biosynthesis pathway of expanding carbon chain for producing advanced biofuels using 2-keto acid intermediates. This biosynthesis pathway can be subdivided into three distinct sub-pathways (I, II, III), each of which results in a corresponding and structurally distinct advanced biofuel. **a** The I sub-pathway is that the carbon chain is extended via adding two carbon atoms (+ 2) with pyruvate as carbon extension unit; the key enzymes are acetohydroxy acid synthase (AHAS) and IlvIHCD. **b** The II sub-pathway is that the carbon chain is lengthened via adding one carbon atom (+ 1) with CoA-dependent molecule as carbon extension unit. Advanced biofuels with a branched chain are obtained via integrating the following natural biosynthetic pathways: α-keto acid-based pathway and CoA-dependent extension pathway.** c** Carbon chain extension of the Type III sub-pathway also occurs involving the addition of one carbon atom (+ 1) using CoA-dependent molecule as the carbon extension unit. Advanced biofuels without branched chains were obtained via utilizing this subtype pathway and conducting a condensation reaction with the overexpression of leuABCD. Green arrows represent using pyruvate as carbon extension unit. Blue arrows represent using CoA-dependent molecule as carbon extension unit. Red arrows represent exogenous decarboxylation and reduction. *ilvA* threonine deaminase; *ilvC* acetohydroxy acid isomeroreductase; *ilvD* dihydroxy acid dehydratase; *ilvGM* acetohydroxybutanoate synthase; *kivd* ketoisovalerate decarboxylase; *leuA*, 2-isopropylmalate synthase; *leuB* 3-isopropylmalate dehydrogenase; *leuCD* 2-isopropylmalate isomerase; *thrA* aspartate kinase, homoserine dehydrogenase; *thrB* homoserine kinase; *thrC* threonine synthase; *Ac-CoA* acetylCoA; *ACP* acyl carrier protein; *CoA* coenzyme A; *OAA* oxaloacetate; *ADH2* alcohol dehydrogenase; *alsS* acetolactate synthase; *asd* aspartate semialdehyde dehydrogenase; *aspC* aspartate aminotransferase; *cimA* citramalate synthase. KIC:2-ketoisocaproate, precursor to l-leucine; *KMV* 2-keto-3-methylvalerate, precursor of l-isoleucine; *PaaH1* β-ketothiolase (BKtB)-3-hydroxy-acyl-CoA dehydrogenase; *Crt* crotonase; *Ter* trans-enoyl-CoA reductase; *adhE* alcohol dehydrogenase. The red arrow shows the oxidation–reduction pathway to higher biofuel. The green arrow is the carbon chain extension pathway

Some natural precursors derived from different intermediates of 2-keto acids metabolic pathways are transformed into advanced biofuels with molecular structure similar to the intermediates based on the ED strategy (Table 1). In this study, the various kinds of 2-keto acids pathways were divided into three sub-pathways (Type I, II, III). Correspondingly, each of the sub-pathways was reviewed and was able to produce distinct advanced biofuels (Fig. 4).

**Table 1:** Advanced biofuels using 2-keto acid intermediates

Advanced biofuels	Producing strain	Key gene from heterologous hosts		Biosynthetic pathways	Key intermediates
Isobutanol	*E. coli * [146], *C. glutamicum* [146]*,* *C. crenatum* [17]*, Bacillus subtilis* [146]*, Brevibacterium flavum* [147]* and S. cerevisiae* [148]	*IlvC, IlvD Kivd,adh2*	*B. subtilis, S. cerevisiae*	Type I	2-Ketoisovalerate
2-Methyl-1-butanol	*E. coli* [8]*,* *C. crenatum* [32]*,* *C. glutamicum* [21]* and S. cerevisiae* [22]	*IlvC,IlvD;Kivd,adh2*	*B. subtilis*	Type I	2-Keto-3-methylvalerate
3-Methyl-1-butanol	*E. coli* [149]*,* *C. crenatum* [23]*, cyanobacteria* [150]* and S. cerevisiae* [151]	*IlvC,IlvD;Kivd,adh2;LeuABCD*	*B. subtilis, S. cerevisiae*	Type II	2-Keto-4-methyl- valerate
4-Methyl-1-pentanol	*E. coli* [152]*, S. cerevisiae* [153]	*IlvC, IlvD;Kivd,adh2;* *LeuABCD*	*B. subtilis,* *S. cerevisiae*	Type II	2-Keto-5-methyl-hexnoate
3-Methyl-pentanol	*E. coli* [23]*,* *S. cerevisiae* [154]	*IlvC, IlvD;Kivd,adh2;* *LeuABCD*	*B. subtilis,* *S. cerevisiae*	Type II	2-Keto-5-methyl-hexnoate
5-Methyl-heptanol	*E. coli* [107]*,* *S. cerevisiae* [23]	*IlvC,,IlvD;Kivd,adh2;* *LeuABCD*	*B. subtilis,* *S.cerevisiae*	Type II	2-Keto-6-methyl-octanoate
Propanol	*E. coli* [155]*,* *C. glutamicum* [156]*,* *S. cerevisiae* [157]	*IlvC, IlvD;Kivd, adh2;* *LeuABCD*	*B. subtilis, S. cerevisiae*	Type III	2-Ketobutyrate
Butanol	*E. coli* [27]*,* *S.cerevisiae* [158]*,* *C. acetobutylicum* [159]*,C.beijerinckii* [160]	Acetyl-CoA acetyltransferase, 3-hydroxybutyryl-CoA dehydrogenase,crotonase,butyryl-CoA dehydrogenase	*B. subtilis, S. cerevisiae*	Type III	2-Ketovalerate
Pentanol	*E. coli* [161]*,* *S. cerevisiae* [162]	*IlvC,IlvD;Kivd,adh2;* *LeuABCD*	*B. subtilis, S. cerevisiae*	Type III	2-Ketocaproate
Hexanol	*E. coli* [163, 164]*,* *S. cerevisiae* [163]	*IlvC,IlvD;Kivd,adh2;* *LeuABCD*	*B. subtilis, S. cerevisiae*	Type III	2-Ketoheptanoate
Heptanol	*E. coli* [165]*,* *S. cerevisiae* [166]	*IlvC,IlvD;Kivd,adh2;* *LeuABCD*	*B. subtilis, S. cerevisiae*	Type III	2-Ketooctanoate
Octanol	*E. coli* [167, 168]*,* *S. cerevisiae* [169]	*IlvC,IlvD;Kivd,adh2;* *LeuABCD*	*B. subtilis, S. cerevisiae*	Type III	2-Ketononaoate

The biosynthetic pathways for Type I sub-pathway was featured by the addition of two carbon number (+ 2) using pyruvate molecules as the carbon chain extension factor. Various advanced biofuels were obtained using this metabolic pathway via the conversion of interlinked intermediates of the pathway (Fig. 4a). Carbon chains specifically, two pyruvate molecules were first concatenated, and then converted into a key intermediate 2-ketoisovalerate (KIV, precursor to l-valine), which was then catalyzed by the catalytic reactions of two catalyzing enzymes: *IlvC,* and *IlvD* [19, 20]. Further extension of the carbon chains could be achieved using this sub-pathway via a condensation reaction between KIV and pyruvate molecule. For example, 2-keto-3-methyl-valerate (KMV) was synthesized and further converted into 2-methyl-1-butanol by an extrinsic catalytic pathway, which involved an oxidation–reduction reaction responsible for generating decarboxylase and reductase [8, 21, 22].

The biosynthetic pathways for Type II was characterized by the extension of one carbon number (+ 1) using CoA-dependent molecule as the carbon extension factor. The advanced biofuels produced via this pathway were primarily alcohols with branched chains that were the result of comprehensive interlacement with other inherent pathways: a natural α-keto acid metabolic pathway and a metabolic pathway that shared characteristics of the Type I sub-pathway (Fig. 4b). It is worth noting that this metabolic pathway could produce various alcohols. Specifically, the intermediate KMV was further converted into 2-keto-4-methyl hexanoate (K4MH, precursor to 3-methyl-pentanol) via the overexpression of a series of enzymes such as LeuA, LeuB, and *Leu*CD [23], using acetyl-CoA as the carbon extension unit [24]. Similarly, other intermediates could be obtained from this metabolic pathway, including 2-keto-5-methyl heptanoate (K5MH, precursor to 4-methyl-hexanol) and 2-keto-6-methyl octanoate (KMO, precursor to 5-methyl-heptanol). These studies showed that carbon chain elongation could be implemented by leveraging the catalytic reaction of *Leu*ABCD, and some substrates of advanced biofuels, such as KMV, K4MH, K5MH, and KMO, could be produced by the metabolic pathway. Subsequently, the production of some branched alcohols with a range of 6 to 10 carbon atoms could be achieved via oxidation–reduction reactions.

The biosynthetic pathways for Type III sub-pathway were also defined by the elongation of one carbon atom number (+ 1) using CoA-dependent molecule as carbon chain expanding factor. The similarity between this sub-pathway and the Type II sub-pathway is that they both leveraged acetyl-CoA as the carbon chain elongation factor, and resulted in a condensation reaction by overexpressing *Leu*ABCD. A distinguishing feature between these two metabolic pathways could be found in the advanced biofuels (C ≥ 6) produced from the Type III sub-pathway as it did not contain any branched chains (Fig. 4c). The Type III sub-pathway could be divided into three steps: amino acid synthesis reaction, deammoniation reaction, and condensation reaction. For example, intermediates 2-ketovalerate (2 kV, precursor to norvaline biosynthesis) were first produced from 2-ketobutyrate (2 KB) via the overexpression of *Leu*ABCD [25]. Then, a variety of precursors of advanced biofuels (C ≥ 6) could be obtained by converting 2 kV intermediates. Finally, 2-ketocaproate (2KC, precursor to *n*-pentanol) were synthesized via a condensation reaction between 2 kV and acetyl-CoA. Based on this strategy, other precursors could produce their corresponding alkane-based alcohols. These precursors include 2-ketoheptanoate (2KH, precursor to *n*-hexanol), 2-ketooctanoate (2KO, precursor of *n*-heptanol), and 2-ketononaoate (2KN, precursor to *n*-octanol), which, similar to 2 kV, could also be obtained via overexpressing *Leu*ABCD [26]. This sub-pathway is significantly less prevalent in natural microbial metabolic systems, with the exception of species from the genus *Clostridium*, which possess an acetone–butanol–ethanol (ABE) pathway [27, 28]. In this strategy, the expansion of carbon chain is derived from the biological catalytic process in which butyryl-CoA is generated from the condensation reaction between two acetyl-CoA molecules via four enzymatically catalyzed steps: acetyl-CoA acetyltransferase, 3-hydroxybutyryl-CoA dehydrogenase, crotonase, and butyryl-CoA dehydrogenase [29–31].

Thus far, researchers have leveraged 2-keto acid intermediates as a biosynthesis strategy for producing various advanced biofuels with longer carbon chain. For example, the metabolic pathway of generating 3-methyl-1-pentanol using *E.coli* is derived from the Type II sub-pathway and requires two catalytic reactions[3, 32]: a carbon chain expansion reaction featuring catalyzing enzymes *Leu*ABCD and a decarboxylation–reduction reaction involving 2-ketoisovalerate decarboxylase (*Kivd*) and alcohol dehydrogenase 2 (*adh*2). Meanwhile, advanced biofuels of unbranched alkane-based alcohols such as *n*-hexanol [33], which could also be generated from 2-keto acid intermediates through the Type II sub-pathway and it involved two important catalytic reactions featuring high-activity enzymes PaaH1, *Crt*, *Ter* in tandem, and an exogenous decarboxylation–reduction reaction: *kivd-adhE* (Fig. 4). 

A large number of studies have proved that 2-isopropylmalate synthase (IPMS) and acetohydroxyacid synthase (AHAS) are two key enzymes involved in the expansion of carbon chains in the biosynthesis strategy that leverages 2-keto acid intermediates. In recent years, in order to improve the efficiency of expanding carbon chains, different carbon–carbon bond transition states catalyzed by *LeuA* mutants (LeuA*) were simulated using quantum mechanics and protein–substrate complexes to identify the best conformers [34]. A diverse range of advanced biofuels with longer carbon chain ranging from 6 to 9 carbons, such as heptanol and octanol, could be produced using this biosynthesis strategy [35, 36]. Therefore, this biosynthesis strategy is a promising alternative for creating a wider variety of advanced biofuels in place of those generated by the fossil industry.

### Production of advanced biofuels using reverse β-oxidation intermediates

Biosynthesis strategy based on reverse β-oxidation intermediates has been demonstrated to show significant promises for expanding carbon chains to produce a variety of advanced biofuels (Table 2), such as various isobutanol and butanol from renewable resources like organic waste [37]. There are some common interactive intermediates between the metabolic pathways that rely on reverse β-oxidation intermediates and those that depend on 2-keto acid intermediates. Especially, the bacterial reverse β-oxidation intermediates have been well studied due to their typical functions in fatty acid catabolism, for which a balance could be maintained with acetyl-CoA as an energy source [38].

**Table 2:** Advanced biofuels using reverse β-oxidation intermediates

Advanced biofuels	Producing strain	Key gene from heterologous hosts		Biosynthetic pathways	Key intermediates
1-Hexanol	*E. coli* [170]*,* *S. cerevisiae* [171]	*yiaY, eutG, and betA,ACoAR,ADH*	*S. cerevisiae*	β-oxidation	2-Ketoisovalerate
1,3-Diols	*E. coli* [172]	*fadB,bktB, phaB,NADPH-dependent acetoacetyl-CoA reductase,butyraldehyde dehydrogenase*	*Megasphaera elsdenii, Ralstonia eutropha*	β-oxidation	Trans-3-hydroxyacyl-CoA
Butanol	*E. coli* [27]*, S. cerevisiae* [173]	Enoyl-CoA hydratase	*S. cerevisiae,* *Clostridium saccharoperbutylacetonicum,*	β-oxidation	2-Keto-4-methylvalerate

**Fig. 5:**
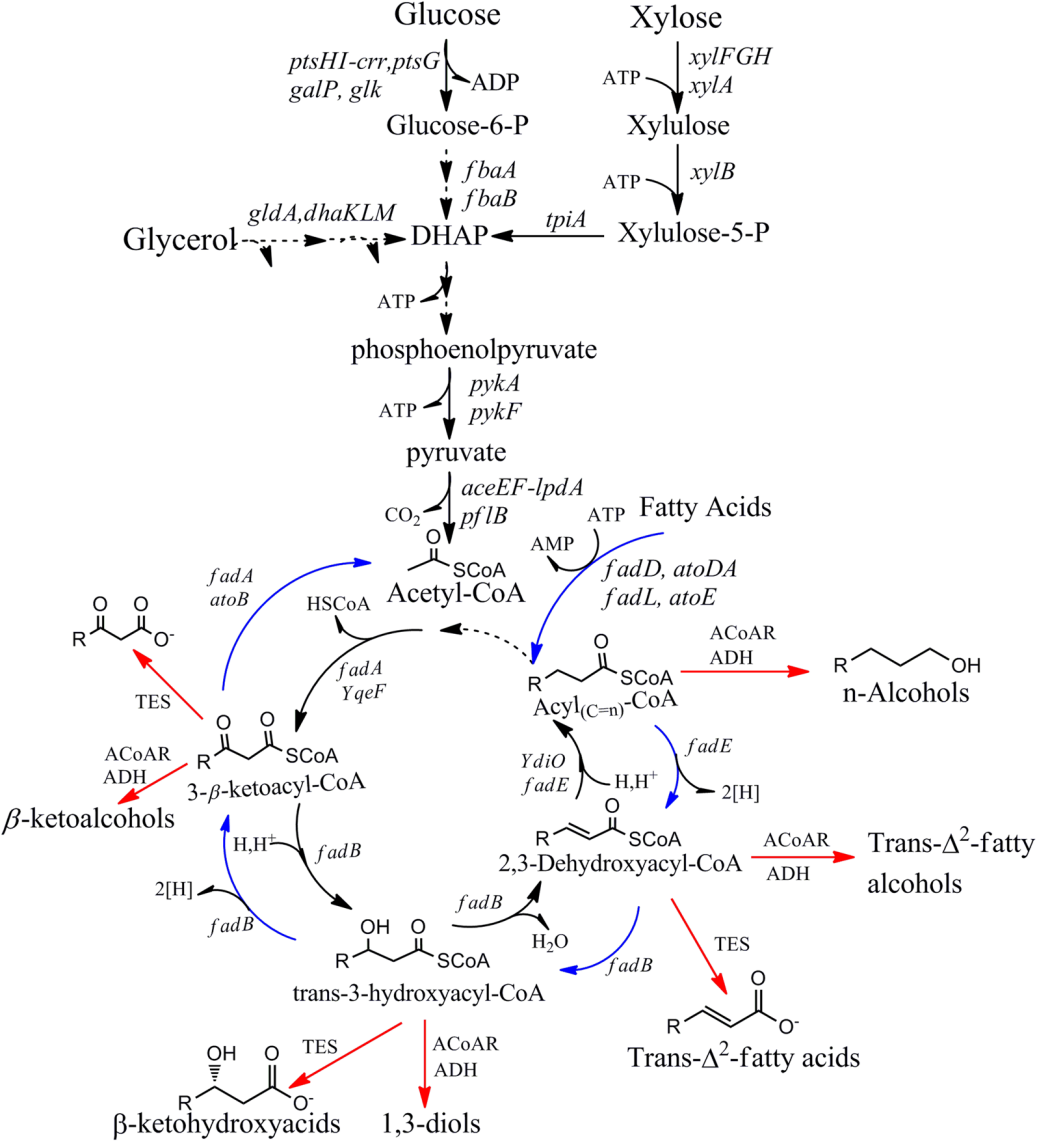
Biosynthesis pathway of expanding carbon chain for producing advanced biofuels using reverse β-oxidation intermediates. This biosynthesis pathway can use acetyl-CoA as a carbon chain elongation factor. Normal carbon chain extension in microorganisms occurs when (Cn + 2)-acyl-CoA molecules are broken down into acetyl-CoA and (Cn)-acetyl CoA molecules under the control of four genes (atoB, fadA, fadB, and fadE). However, chain elongation occurring through the addition of acetyl-CoA to another thioester due to β-oxidation cycle metabolic pathway is essentially reversible. Thus, advanced biofuels could be produced after one turn of this cycle. *YqeF* acetyl-CoA acetyltransferase; *fadA* ketoacyl-CoA thiolase; *fadB* hydroxyacyl-CoA dehydrogenase and enoyl-CoA hydratase; *YdiO* enoyl-CoA reductase; *TES* thioesterase; *AcoAR* acylCoA reductase; *ADH* alcohol dehydrogenase; *aceEF-lpdA* pyruvate dehydrogenase multi-enzyme complex; *acetyl-CoA* acetoacetyl-CoA transferase; *atoB* acetyl-CoA acyltransferase; *atoE* putative short-chain fatty acid transporter; *dhaKLM* PEP-dependent dihydroxyacetone kinase; *fadA* 3-ketoacyl-CoA thiolase; *fadB* enoyl-CoA hydratase/3-hydroxyaceyl-CoA dehydrogenase; *fadD* fatty acyl-CoA synthetase; *fadE* acyl-CoA dehydrogenase; *fadL* long-chain fatty acid outer membrane transporter; *fbaA and fbaB*: fructose-1,6-bisphosphate aldolase; *fdhF* formate dehydrogenase; *galP* galactose permease; *gldA* glycerol dehydrogenase; *glk* glucokinase; *glpF* glycerol MIP channel; *hycB-1* hydrogenase; *pflB* pyruvate formate lyase; *ptsHI-crr: ptsG* glucose specific phosphoenolpyruvate phosphotransferase system; *pykA and pykF* pyruvate kinase; *tpiA* triose phosphate isomerase; *xylA* xyloseisomerase; *xylB* xylulokinase; *xylE* xylose MFS transporter; xylFGH: xylose ABC transporter. 2[H]: NAD(P)H = FADH2; *DHA* dihydroxyacetone, *DHAP* dihydroxyacetone-phosphate; Fructose-1: 6-BP fructose-1,6-bisphosphate. The red arrow shows the oxidation–reduction pathway to higher biofuel. The blue arrow is the carbon chain extension pathway

Acetyl-CoA is used as the elongation factor of the reverse β-oxidation intermediates (Fig. 5). The process of expanding carbon chains involves the decomposition of intermediates (C_n+2_)-acyl-CoA into two main compounds, acetyl-CoA and (C_n_)-acetyl CoA intermediates, via a series of catalytic reactions of the four catalyzing enzymes *atoB*, *fadA*, *fadB*, and *fadE*. A circulation process in which two carbon molecules from the fatty acid pathway are removed through the β-oxidation cycle, followed by the addition of one acetyl-CoA molecule to the tricarboxylic acid (TCA) cycle (Fig. 5). The carbon chain could be further elongated since the acetyl-CoA is added to another thioester due to the reversibility of the β-oxidation metabolic cycle. Finally, advanced biofuels could be produced after one round of this cyclic process. For example, to produce advanced biofuel 1-butanol, acetoacetyl-CoA is first obtained via a condensation reaction of two acetyl-CoA molecules by acetyl-CoA C-acetyltransferase. Subsequently, 3-hydroxybutyryl-CoA is obtained through 3-hydroxyacyl-CoA dehydrogenase or enoyl-CoA hydratase to reduce the β-keto acid products, which are subsequently converted into crotonyl-CoA. Then, crotonyl-CoA is hydrogenated and converted into butyryl-CoA, thereby, adding a carbon to the carbon chain. The butyryl-CoA is converted into 1-butyraldehyde by a CoA-dependent aldehyde dehydrogenase. Finally, 1-butanol is produced from 1-butyraldehyde via a reduction reaction using *adhE*. Carbon chain extension could be achieved by consecutive β-oxidation process, from which advanced biofuels with longer carbon chains could be produced [39, 40].

Previous studies have demonstrated that advanced biofuel synthesis could be influenced by fatty acid accumulation [41]. Under low fatty acid conditions, the catalytic enzymes in the reverse β-oxidation process could convert some intermediates from the fatty acid metabolic pathway into a diverse collection of alcohols with long carbon chain. Due to the cyclical nature of this biosynthesis strategy, the length of carbon chain and the degree of saturation of the metabolic intermediates could be controlled by selecting the appropriate terminal monospecific enzyme [42–44]. In addition, extensive modification of the carbon chain is feasible with this reverse β-oxidation intermediates by reducing fatty acid degradation, removing other carbon metabolic flux in the circuit branch, and avoiding feedback inhibition. Furthermore, reverse β-oxidation intermediates have been used to produce a variety of higher-order advanced biofuels including advanced fatty alcohols with 6–10 carbon atoms such as 1-hexanol, 1-octanol, and 1-decanol [45, 46].

Although the biosynthesis strategy based on reverse β-oxidation intermediates could be used to expand carbon chain in a manner that resembles the sequence of CoA-dependent reactions, the catalytic mechanism involved has yet to be thoroughly studied. Even so, advanced biofuels including some alcoholic compounds with long carbon chain (C6–C10) have been produced using reverse β-oxidation intermediates. For example, 1-hexanol at a yield of 37 mg/L was obtained by expressing alcohol dehydrogenases *yiaY*, *eutG*, and *betA* [15, 47] and expressing open reading frames ydiO and ydiQRST encoding components of the acyl-CoA dehydrogenase complex of anaerobic fatty acid β-oxidation [48]. However, it is unclear whether electron transfer flavoproteins and ferredoxin (YdiQRST) exert any important effects on the catalytic process [49, 50].

Nevertheless, a variety of fatty acids and alcohols with various carbon chain could be achieved by this approach due to the interaction between 2-keto acids and the β-oxidation process [51, 52]. Therefore, this biosynthesis strategy is considered to have great potential for the synthesis of advanced biofuels.

### Production of advanced biofuels using fatty acid intermediates

Biodiesel fuel, as an advanced biofuel, has been proposed as a potential substitute for petroleum-based diesel. Biodiesel fuel is typically produced from fatty acid methyl esters (FAMEs) or fatty acid ethyl esters (FAEEs) via the conversion of plant or animal oils. Decarboxylated derivatives, mainly fatty alcohols, with long aliphatic chains or aromatic hydrocarbon molecules can also be synthesized via the transesterification of acylglycerols using this biosynthesis strategy based on fatty acid intermediates. For example, free fatty acids (FFAs) from *cyanobacteria* can be converted into biodiesel, alcohols, alkanes, and olefins with long carbon chain [53, 54], which are dependent on highly efficient catalytic enzyme in order to achieve esterification. It is well-known that eukaryotic algae, such as photosynthetic microorganisms and yeast *S. cerevisiae* can also naturally produce a great quantity of acylglycerols, which can be subsequently converted into various advanced biofuels [55–57]. However, studies have shown that it is rare for bacteria to naturally generate large quantities of FAMEs and FAEEs. Therefore, using bacteria as the engineering strains for expanding carbon chains to synthesize advanced biofuels based on FAMEs or FAEEs production is of key interest. In this study, two biometabolic pathways using fatty acid intermediates were reviewed according to the production of different kinds of advanced biofuels.

#### Production of advanced biofuels: fatty alcohols using fatty acid intermediates

**Fig. 6:**
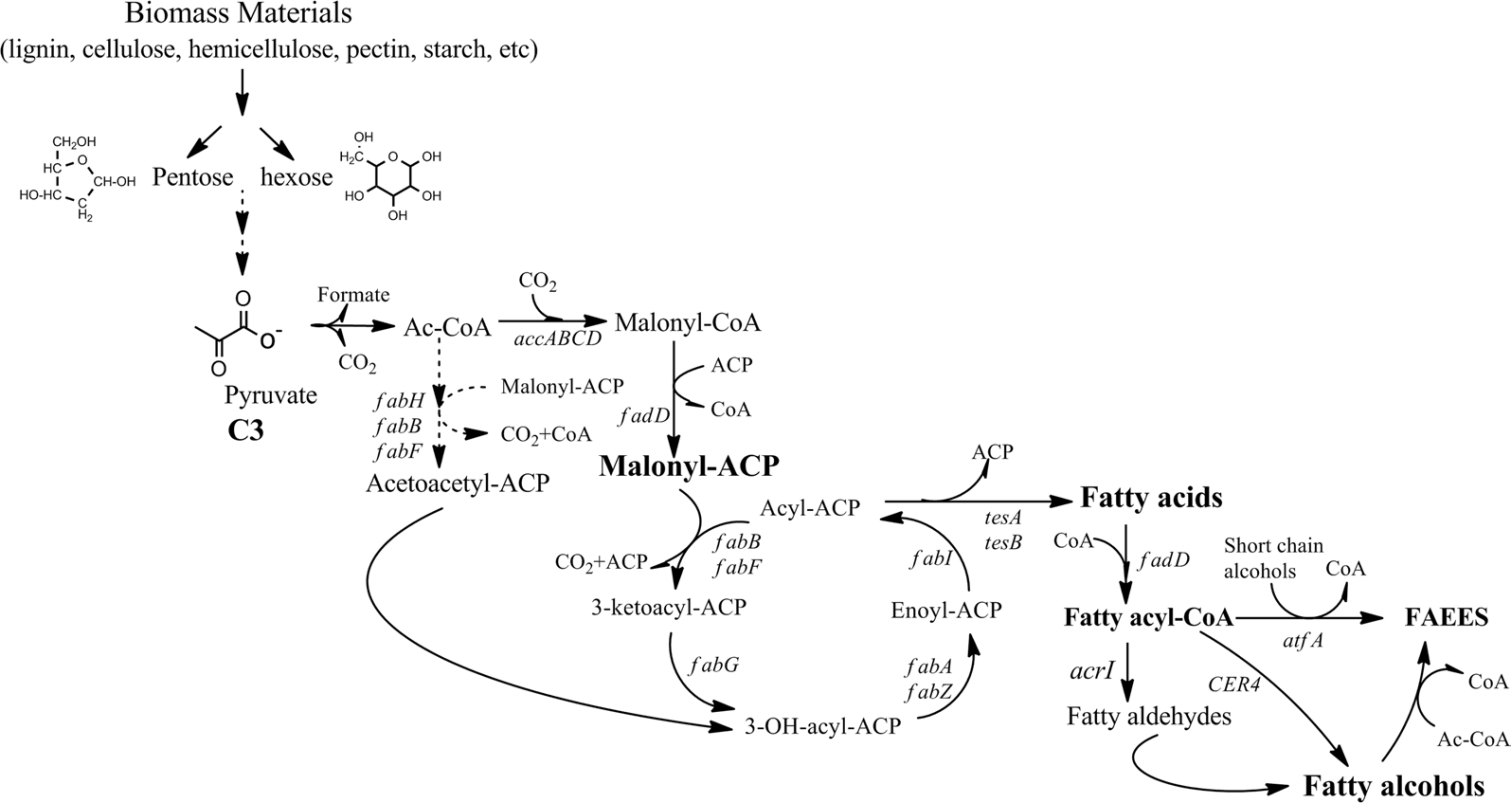
Biosynthesis pathway to produce advanced biofuels of fatty alcohols using fatty acid intermediates. Advanced biofuels: fatty alcohols product from carbon chain extension of fatty acid synthesis. Mechanistically, carbon chain extension relies on acetyl-CoA as the primary carbon source in the fatty acid synthetic pathway via ATP-dependent carboxylation into malonyl-CoA. Acyl units are then provided for carbon elongation by cycles of decarboxylative addition of malonyl-CoA. *accABCD* acetyl-CoA carboxylase; *acrI* acyl-CoA reductase; *asd* aspartate semialdehyde dehydrogenase; *atfA* acyltransferase; *fabA* hydroxydecanoyl-ACP dehydrase; *fabB* ketoacyl-ACP synthase I; *fabD* malonyl-CoA-ACP transacylase; *fabF* ketoacyl-ACP synthase II; *fabG* ketoacyl-ACP reductase; *fabH* ketoacyl-ACP synthase III; *fabI* enoyl-ACP reductase; *fabZ* hydroxyacyl-ACP dehydratase; *CER4* fatty acyl-CoA reductase acyl-CoA synthetase; *FAEE* fatty acid ethyl ester; *Ac-CoA* acetylCoA; *ACP* acyl carrier protein; *CoA* coenzyme A

Expanding carbon chain to produce non-natural fatty alcohols needs to use acetyl-CoA as the original carbon source in the FADs (Table 3). In some microorganisms, FFAs with long carbon chain from malonyl-CoA are synthesized using ATP-dependent carboxylation. Acyl units could also be added during elongation of carbon chain due to the decarboxylation effect of malonyl-CoA (Fig. 6). For example, acetyl-CoA in *E. coli* is first converted into malonyl-CoA under the actions of ATP-dependent-acetyl-CoA carboxylase (AccABCD). The FFA compound bound to an acyl carrier protein (ACP) is then produced through a NADPH-dependent reaction in which malonyl-CoA is converted by a multicomponent fatty acid synthase. These fatty acyl-ACPs are utilized as either carbon chain elongation structures or energy storage molecules. The FFAs are generated from these energy stores, and structural components can be reconstructed by overexpressing thioesterases that cleave fatty acyl-ACPs. Cleavage reaction of fatty acyl-ACP can lead to the accumulation of large amounts of fatty acyl-CoAs because the gene cytoplasmic (normally periplasmic) thiolase can hydrolyze fatty acyl-ACPs by fatty acid transport and *fadDE* gene. Finally, the FFAs with long carbon chain are added to some alcohols with short carbon chains between C8 and C18 under the action of different thioesterases (Fig. 6). For example, the fatty acyl-CoAs obtained from the above reactions can be modified into C12–C18 fatty alcohols in *Saccharomyces cerevisiae and Escherichia coli* [58, 59] by importation of a reductive pathway using fatty acyl-CoA reductase from other microorganisms such as *Acinetobacter calcoaceticus* [5, 58, 59].

**Table 3:** Advanced biofuels: fatty alcohols using fatty acid intermediates

Advanced biofuels	Producing strain	Key gene from heterologous hosts		Biosynthetic pathways	Key intermediates
Long chain alcohols	*E. coli* [174]*,* *S. cerevisiae*[175]	*atfA*	*S. cerevisiae,* *Paracoccus denitrificans*	Fatty alcohols using fatty intermediates	FAEES
C12–C18 fatty alcohols	*E. coli* [176, 177]*,* *S. cerevisiae*[178]	acyl-CoA reductase	A. *calcoaceticus*	Fatty alcohols using fatty intermediates	Fatty aldehydes
C10–C18 fatty alcohols	*E. coli* [179]*,* *S. cerevisiae* [180]	*CER4*	*S. cerevisiae*	Fatty alcohols using fatty intermediates	Fatty acyl-CoA
1,3-Diols	*E. coli* [181]*,* *S. cerevisiae* [182]	*acrI*	*S. cerevisiae*	Fatty alcohols using fatty intermediates	Fatty acyl-CoA

Previous literature has demonstrated that yield of fatty alcohols can be increased via systemic improvement of metabolic genes involving fatty acid intermediates. For example, some studies have shown that deletion of *fadD*, overexpression of AccABCD genes, and heterologous expression of ACP thioesterase can successfully increase FFA levels in an *Umbellularia californica* plant, resulting in enhanced fatty acids production. Some reports show an effective approach that leveraged plasmids containing different copy numbers and collections of acyl-CoA ligase, thioesterase, and acyl-CoA reductase from various biospecies were reported for the optimization of fatty acids production. For example, *E. coli* and *Saccharomyces* were optimized using this biosynthesis pathway, which produced fatty alcohol at a yield of 0.81 mg/L with 12-carbon chain when glycerol was used as the substrate. In addition, a series of fatty acids with different lengths of carbon chain (C10–C18) and levels of saturation were also generated based on this biosynthesis pathway using *E. coli* [60–62].

#### Production of advanced biofuels: alkanes, alkenes and ketones using fatty acid intermediates

Alkanes and alkenes are the most important advanced biofuels that are used as substitutes for hydrocarbon fossil fuel such as jet fuels, diesel, and gasoline. With recent advances in system metabolic engineering, synthetic mechanisms of alkanes and alkenes have been investigated in depth to allow for a better understanding of their underlying metabolic pathways (Table 4). There are currently two metabolic pathways for producing alkanes and alkenes, namely elongation–decarboxylation [63, 64] and head-to-head condensation [65, 66], both of which rely on fatty acid intermediates for carbon chain expansion. The elongation–decarboxylation pathway uses acyl-CoA from fatty acid intermediates as an extension factor via the cyclic addition of the two-carbon molecules from the conversion of malonyl-CoA, followed by subsequent decarboxylation to produce alkanes and alkenes. The head-to-head condensation pathway involves the conjugation of two fatty acid derivatives that are converted into carboxylic acid via Claisen condensation, from which alkenes with odd carbon chain are subsequently generated by decarboxylation and reduction reactions [25, 67–69].

**Table 4:** Advanced biofuels: alkanes, alkenes and ketones using fatty acid intermediates

Advanced biofuels	Producing strain	Key gene from heterologous hosts		Biosynthetic pathways	Key intermediates
Alkanes and alkenes (C13–C17)heptadecane, heptadecene, tridecane,pentadecene,pentadecane,heptadecene,pentadecane, heptadecane	*E. coli* [62]*, Synechocystis sp. PCC6803* [36, 183]	ADO,AAR	*Synechococcus sp. PCC7002*	AAR-ADO	Fatty aldehyde
Linear alkanes, such as pentadecane,heptadecene,branched tridecane, heptadecane, hexadecane, hexadecane, pentadecane, pentadecene, tridecane, tridecene, undecane	*E. coli* [184]*, Synechococcus sp. PCC 7002* [185]* and cyanobacterium Lyngbya majuscula* [186]	CAR/Sfp FAR;luxC, luxE, and luxD	*Mycobacterium marinum and Bacillus subtilis,Photorhabdus luminescens*	CAR/FAR-ADO	Fatty acid
α-Olefin (C16–C20) 1,10-heptadecadiene and 1-pentadecene	*Jeotgalicoccus sp. ATCC 8456* [187]	OleTJE	*Jeotgalicoccus sp. ATCC 8456*	OLeTJE	Acyl-CoA
Olefin	*Micrococcus luteus* [76]	OleABCD	*Micrococcus*	HEAD-TO-HEAD	Acyl-CoA
1-Nonadecene,1,14-nonadecadiene	*cyanobacterium Lyngbya majuscula* [188]	ols gene	*Synechococcus sp. PCC 7002 and cyanobacterium Lyngbya majuscula*	OLS pathway	Acyl-CoA
Isooctane	*Streptomyces albus* [84]	type I polyketide synthase	*Synechococcus sp. PCC 7002*	Polyketone synthesis pathway	Acyl-CoA

**Fig. 7:**
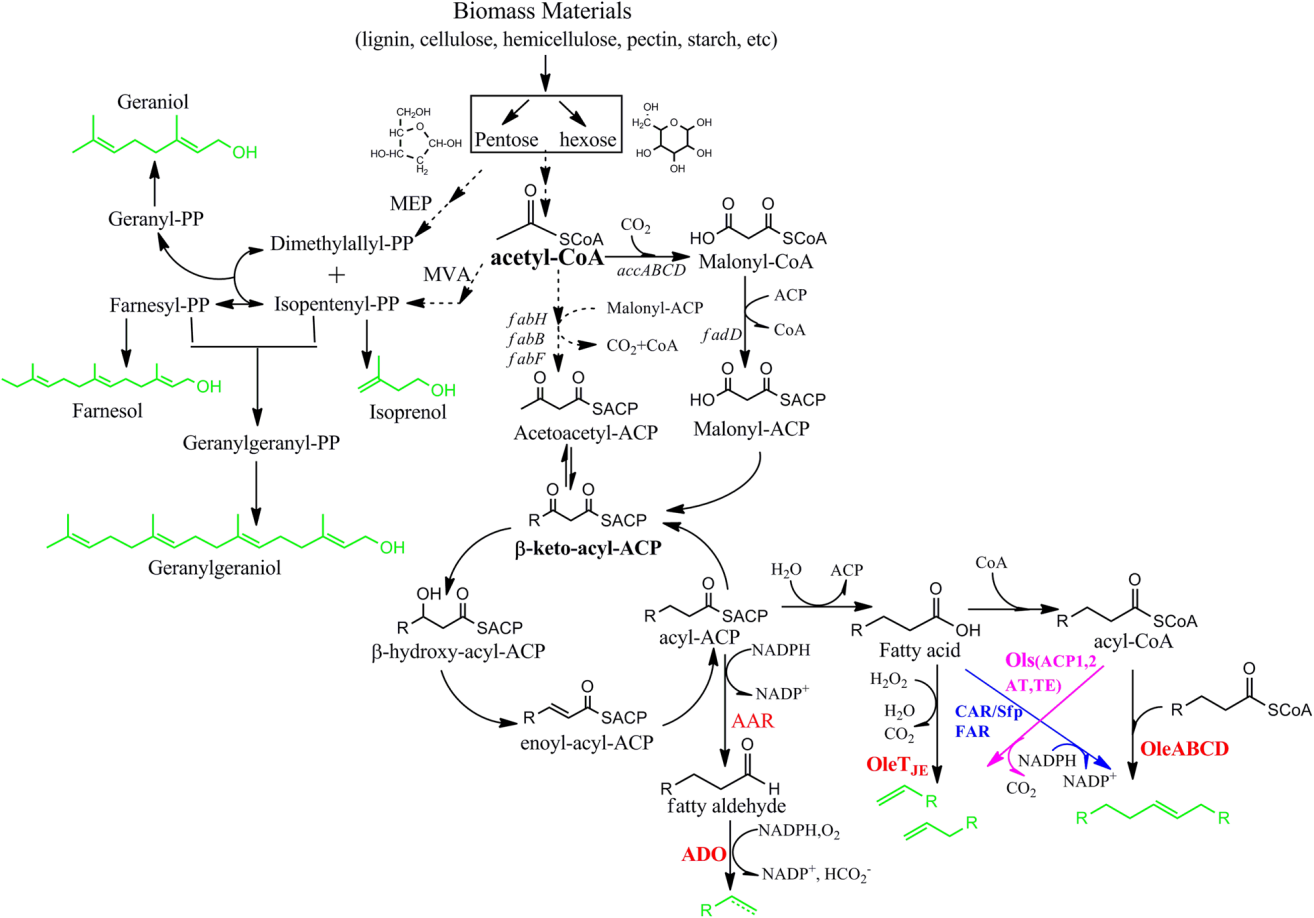
Biosynthesis pathway to produce advanced biofuels of alkanes and alkenes using fatty acid intermediates. Increasing microbial synthesis of alka(e)nes via carbon chain extension of five different fatty acid synthesis pathways that can convert free fatty acids or fatty acid derivatives into alka(e)nes, classified as “elongation–decarboxylation” and “head-to-head condensation” biosynthetic metabolic approaches, including AAR-ADO, HEAD-TO-HEAD, OLeTJE, OLS, and CAR/FAR-ADO pathways. The elongation–decarboxylation approach is based on the use of acyl-coenzyme A (CoA) from a fatty acid synthesis pathway as an extension factor for carbon chain elongation via the circular addition of a two-carbon unit, conversion of malonyl-CoA, and subsequent decarboxylation to produce chain alka(e)nes. The second approach is head-to-head condensation, involving the conjugation of two fatty acid derivatives to a carboxylic acid via a Claisen condensation. The odd chain alka(e)ne is formed by decarboxylation and decarbonylation. *ACP* acyl–acyl carrier protein; *ACC* acetyl-CoA carboxylase; *FabD* malonyl-CoA ACP transacylase; *FabH* β-keto-acyl-ACP synthase III; *FabB* β-keto-acyl-ACP synthase I; *FabG* β-keto-acyl-ACP reductase; FabZ: β-hydroxyacyl-ACP dehydratase; *FabI* enoyl-acyl-ACP reductase; *TE* thioesterase; *FadD* acyl-CoA synthase; *AAR* acyl-ACP reductase; ADO: aldehyde-deformylating oxygenase; OleABCD: a four protein families for long-chain olefin biosynthesis; (OleTJE): fatty acid decarboxylase, a cytochrome P450 enzyme that reduces fatty acids to alkenes; CAR: carboxylic acid reductase; Sfp: A phosphopantetheinyl transferase; FAR: fatty acid reductase; Ols: a type I polyketide synthases for α-olefin biosynthesis. MVA indicates mevalonic pathway for isoprenoid biosynthesis. MEP indicates the methylerythritol phosphate pathway. Red, purple, blue fonts show the oxidation–reduction pathway to higher biofuel

Previous studies have shown that pathways that convert FFAs or fatty acid intermediates into alkanes and alkenes can be defined by five diversity-oriented sub-pathways, including AAR-ADO, HEAD-TO-HEAD, OLeT_JE_ OLS, and CAR/FAR-ADO (Fig. 7). For AAR-ADO, two very important catalytic enzymes from *Synechococcus sp.* PCC7002, namely AAR and ADO, participate in the synthesis of alkane [70]. The catalytic process involves Acyl-ACP reductase, which can reduce the conversion of ACP into aldehyde small compounds, followed by the conversion of aldehyde into alkane or alkene via ADO [71]. An engineered prokaryote, such as *E. coli* and *Synechocystis* sp. PCC6803, can produce multifarious alkanes and alkenes (C13–C17) that include some advanced biofuels, such as heptadecane, heptadecene, tridecane, pentadecene, pentadecane, heptadecene, pentadecane, and heptadecane, by overexpressing AAR and ADO derived from cyanobacteria strains [72].

Meanwhile, head-to-head condensation reaction [73]can generate advanced biofuels with long carbon chain via the expansion of carbon chain such as olefins (Fig. 7). For example, in *Micrococcus luteus*, olefin synthesis allows for the expansion of carbon chain via the action of four catalytic enzymes *Ole*A, *Ole*B, *Ole*C, and *Ole*D [74]. These four enzymes include an α/β-hydrolase, a thiolase, a short-chain dehydrogenase, and an AMP-dependent ligase or synthetase. Some unsaturated alkenes with long carbon chain, such as 27:3, 27:2, 29:3, and 29:2, can be produced using this sub-pathway [75, 76].

The engineering sub-pathway OL_e_T_JE_ was first identified in the bacteria *Jeotgalicoccus sp*. ATCC 8456 (Fig. 7). The specific case is terminal *n*-alkene can be synthesized via conversion of FFAs into terminal olefins using this strategy [77]. For example, the α-olefin (C16–C20) can be produced through the expansion of carbon chain by direct decarboxylation of FFAs with long carbon chain with fatty acid decarboxylase Ol_e_T_JE_, using H_2_O_2_ as a source of electrons [78]. For example, 1,10-heptadecadiene and 1-pentadecene have been obtained using this engineering strategy [79].

The sub-pathway OLS pathway was first discovered in marine cyanobacterium *Synechococcus sp*. PCC 7002 and cyanobacterium *Lyngbya majuscula*. The *ols* gene is responsible for encoding type I polyketide synthase (PKS), including multi-domain ketosynthase (KS), acyltransferase (AT), and ketoreductase (KR). The expansion process of carbon chain involves the combination of acyl-ACP with the ACP1 domain to form an acyl-substrate. Then, two carbons from malonyl-CoA are added to the acyl-substrate by the central extension module of KS, AT, and KR. Subsequently, the ACP2 domain and β-keto group are reduced to generate β-hydroxyl, which is then activated by sulfotransferase (ST) via sulfation. Finally, the C-terminal of the thioesterase (TE) domain catalyzes dehydration and decarboxylation reactions to produce the advanced biofuels olefin [80]. For example, 1-nonadecene [81] and 1,14-nonadecadiene can be synthesized according to this engineering strategy using curM-encoding enzyme with ORF *ols* as the agent responsible for expressing olefin synthase [82, 83]. Some various short-chain ketones (C5–C7) such as ethyl ketone, methyl ketone can be produced by introducing a hybrid polyketide synthase (PKS) via fusing a PKS subunit of lipomycin synthase LipPks1 and a thioesterase (TE) in Streptomyces hosts [84].

The engineering sub-pathway CAR/FAR-ADO is dependent on three very important catalytic enzymes: carboxylic acid reductase (CAR), fatty acid reductase (FAR), and phosphopantetheinyl transferase (*Sfp*) [85]. The CAR and *Sfp* catalytic enzymes are derived from two bacteria, *Mycobacterium marinum* [86] and *Bacillus subtilis* [87], respectively. The process of expanding carbon chain involves the conversion of FFAs into fatty aldehydes by CAR or FAR complex with the genes luxC, luxE, and luxD isolated from *Photorhabdus luminescens* [88, 89]. After oxidation, the fatty aldehydes are converted into alkanes and alkenes with straight or branched chains. This conversion of fatty aldehydes is responsible for the ADO catalytic reaction by *Sfp* from *Cyanobacteria* in combination with a *B. subtilis* thioesterase, branched chain α-keto acid dehydrogenase complex, lipase, and β-keto-acyl-ACP synthase III [90]. Some linear alkanes, such as pentadecane, heptadecene, branched tridecane, heptadecane, hexadecane, hexadecane, pentadecane, pentadecene, tridecane, tridecene, and undecane can be obtained via this engineering sub-pathway [91–93].

**Fig. 8:**
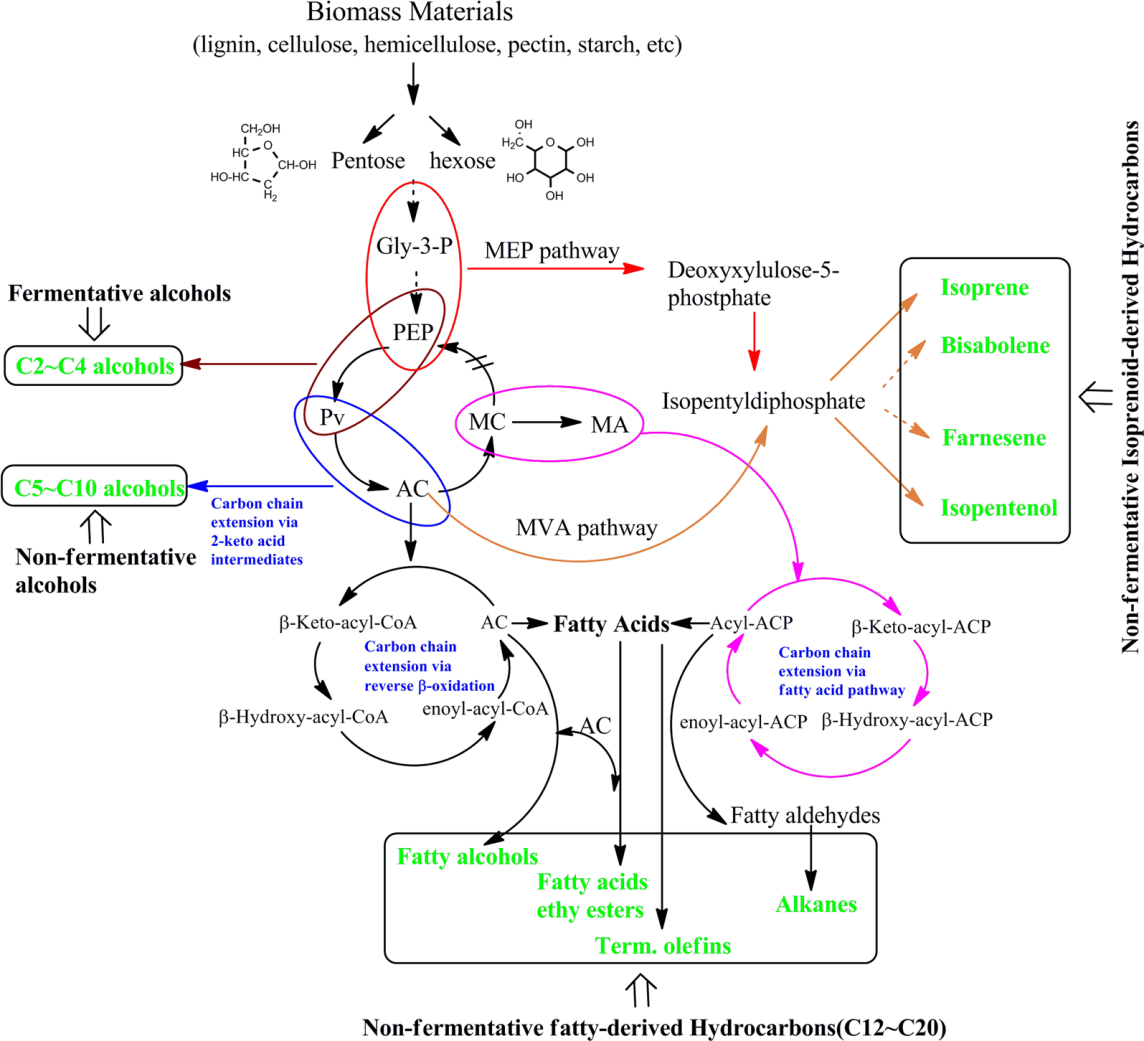
The complex relationship in different biosynthesis pathways of expanding carbon chain. Every one-carbon chain extension generates a global net effect per catalytic cycle regardless of whether the pathway involving 2-keto acid intermediate (“ + 1” or “ + 2” pathway) or a biosynthesis pathway using fatty acid intermediates and reverses β-oxidation. A mixture containing various advanced biofuels namely, longer, branched or unbranched carbon chains (e.g., alcohols) is capable of producing via the per addition of one carbon. Gly-3-P: glyceraldehyde-3-phosphate; *PEP* phosphoenolpyruvate; *Pv* pyruvate; *MC* malonyl-CoA; *MA* malonyl-ACP; *AC* acyl-CoA

In summary, the biosynthesis pathways based on 2-keto acid intermediates, fatty acid intermediates or reverse β-oxidation intermediates could be used to add “ + 1” or “ + 2” carbon molecules to carbon chain. Addition of one-carbon molecule could agitate the whole metabolic network by influencing the process of catalytic reactions (Fig. 8). Therefore, a mixture including various advanced biofuels with longer branched or unbranched carbon chains, such as alkanes, alkenes and alcohols, can be obtained during carbon chain expansion by leveraging specific biometabolic pathways for the addition of each carbon molecule. These pathways are presented as an important tool for the production of aliphatic acids and advanced biofuels due to their ability to selectively lengthen carbon chain via multiple carbon cycles. This exact level of control in the process of carbon chain expansion has been demonstrated using *E. coli* to produce many advanced biofuels such as isobutanol at a yield of 22 g/L [19, 94]. Currently, the engineering strategy with 2-keto acid intermediates is one of the most thoroughly researched strategies that combines other engineering technologies derived from protein-substrate, quantum mechanical, metabonomics, and structural protein.

In the aforementioned biometabolic pathways for expanding carbon chain, CoA molecules are first converted to some intermediates via addition of carbon molecule. For example, synthesis of butanol involves the extension of carbon chain using a series of CoA molecules: acetyl-CoA acetyltransferase, 3-hydroxybutyryl-CoA dehydrogenase, crotonase, and butyryl-CoA dehydrogenase [95]. Therefore, selecting an appropriate engineering strategy is critical for fine-tuning of both the expression of multiple catalytic enzymes and the concurrent adaption of cellular physiology during carbon chain expansion.

## Technical approaches for overcoming low efficiency in carbon chain expansion

In order to overcome the problem of low efficiency, from the early traditional modification techniques such as eliminating the generation pathway of by-products, releasing the synthesis inhibition of products, overexpression of key enzymes in the synthesis pathway, to the development of metabolic response database, and machine learning-based network model calculation, optimal assembly of metabolic pathways, dynamic regulation of gene expression and other new metabolic modification techniques. There are usually the following technical approaches for overcoming low efficiency in carbon chain expansion.

### DNA fragment assembly technology

The creation and optimization of biosynthetic pathways for carbon chain extension first require a breakthrough in DNA fragment assembly technology. DNA fragment assembly technology is an important prerequisite for the creation of biosynthetic pathways for producing biofuel [96]. Traditional DNA assembly relies mainly on restriction endonuclease and DNA ligase. However, this assembly method has certain limitations, such as the “scar” of enzyme restriction site will be left at the junction, and the corresponding enzyme restriction site cannot exist in the gene fragment to be assembled, thus reducing the range of selective endonuclease [97, 98]. The latest technologies include the following three.

1. Golden Gate assembly technique

Restriction endonuclease Type II, which has features that identify and cut the position inconsistencies of double-stranded DNA, is required. This property enables it to cut DNA outside the recognition site, and the sticky ends generated are connected sequentially under the action of ligase and assembled into DNA fragments without the enzyme cleavage site, realizing the seamless connection of multiple fragments. It maybe provide opportunities in creating a flexible, versatile, and data-driven framework to support biofuel research and development in biofoundries [99, 100].

(B) Gibson assembly technique

No restriction enzyme is required, but the ends of each DNA fragment contain homologous sequences of 15-20 bp, according to which the assembly is carried out in the order of homologous sequences. The assembly process involves several enzymes: DNA 5' exonuclease, which first cuts out a sticky terminal, which complicates each other according to homology, and polymerase DNA polymerase, which replicates the missing bases. Finally, the sticky end is attached by DNA ligase. This method allows one or more DNA fragments to be inserted into a linearized vector quickly, efficiently and precisely in a predetermined direction, achieving “seamless” assembly [101]. This technology has been used to design, build, test, learn steps of yeast-based biofuel production [102, 103].

(C) CPEC technique

Circular polymerase extension cloning (CPEC) is a short name for circular polymerase extension cloning. The requirements for DNA fragments are similar to Gibson's, and the ends contain homologous sequences. CPEC is a technology to realize the assembly of DNA fragments by PCR: DNA fragments and vectors are denatured and decomposed, and the end homologous sequences complement each other during annealing. The sequences are mutual templates and primers and extend into circular DNA molecules containing gaps under the action of DNA polymerase [104]. The gaps can be repaired in *E.coli* to obtain complete plasmids. In general, CPEC is a simpler, more efficient and economical DNA assembly technology with more extensive applications, but it is not suitable for the assembly of DNA fragments with high GC content. A standard CPEC protocol 67 was used to assemble the three PCR products to produce biofuel [105, 106].

### Gene expression regulation techniques

Gene expression regulation is an important means to optimize biosynthetic pathways and is the core to overcome the low efficiency of target product production. After the pathway is created in the host, it is necessary to regulate the expression intensity of genes at key nodes, including enhancing the product synthesis pathway and cofactor synthesis pathway, or weakening some genes that will lead to non-growth of bacteria and have adverse effects on product production after knockout. According to the technical level, it can be divided into single gene regulation, polygene regulation and gene dynamic regulation.

1. Single gene regulation

Single gene regulation is to regulate the expression of a specific gene of metabolic pathway on chromosomes. In the early stage of metabolic engineering, strong promoters are commonly used for single gene regulation, such as IPTG-induced Tac promoter and T7 promoter. However, strong expression of single genes may not be optimal for metabolic pathways. To achieve optimal expression of a single gene, promoter libraries in *E. coli* were constructed and to obtain promoters of different strengths for the regulation of glucose transporter gene galP and glucokinase gene *glk* [107, 108]. The combined regulatory strain GalP93-GIk37 significantly increased the rate of glucose consumption. In addition to the promoter library, the regulation of single genes of the succinic acid transporters DcuB and DcuC by RBS library can also improve the ability to transport succinic acid to the cell and increase the yield of the final product 2,3-butanediol [109–111].

(B) Multi-gene regulation

Single-gene regulation can only be operated on the single gene dimension. Even if combined regulation, the library capacity is greatly reduced, and it is difficult to screen out the optimal combination of multiple genes. In the optimization of metabolic pathway, to achieve the efficient production of target biofuel with long-carbon chain, the cooperative expression of multiple genes is often required to achieve the optimization of metabolic pathway. Keasling's team developed a tunable intergenic region (TIGR) library technique [112]. Based on the principle that inter-gene sequence changes affect gene expression intensity, this technique achieves the aim of simultaneously regulating the expression intensity of multiple genes within a single operon [113, 114]. Multiple genes of mevalonate pathway in *E.coli* realized cooperative expression, and mevalonate production was increased by 7 times using this technique [115, 116]. Isaacs et al. developed multiplex automated genome engineering (MAGE) technology, which improves the efficiency of multiplexed gene editing using red homologous recombination system and automatic cycling equipment [117]. Based on the subsequent CRISPR/Cas9 gene-editing technology, three gene libraries of xylose metabolic pathway were regulated on chromosomes, the optimal strain was screened, and the xylose metabolic rate was increased by three times [118, 119].

(C) Gene dynamic regulation

Dynamic regulation is one of the most effective strategies in metabolic pathway optimization and an important technique to improve the target product. Although static regulation such as single- and multigene regulation is easy to operate and effective, the intracellular metabolites are dynamic. If the expression level of related genes is too high, the cell resources will be wasted; if the expression level is too low, the metabolic pathway will be restricted, and the yield and output of the final target products will be difficult to improve due to the imbalance of cell metabolism. In order to timely balance the relationship between gene expression and global metabolism required for product synthesis, the introduction of gene dynamic regulation can respond to metabolic signals in real time, and make timely feedback regulation to adapt to changes in cellular metabolism and environment. For example, the *S. cerevisiae* was engineered to produce and secrete 1-alkenes by manipulation of the fatty acid metabolism, enzyme selection, engineering the electron transfer system and expressing a transporter [120]. Furthermore, a dynamic regulation strategy were implemented to control the expression of membrane enzyme and transporter, which improved 1-alkene production [121–123].

### Genome editing technique

In order to improving microbial synthesis efficiency, through gene editing, exogenous gene insertion, endogenous gene knockout and key gene expression regulation can be realized for expanding carbon chain. Homologous recombination system is the basis of bacterial and fungal genome editing technology [124, 125]. The most commonly used system is the red homologous recombination system, which is derived from phage and includes three proteins, Exo, Beta and Gam, and achieves homologous recombination of foreign fragments with the coordination of these three proteins [125]. Homologous recombination is widely used in *E.coli* and other microorganism gene editing for producing biofuels [126]. Based on the Red homologous recombination system and combining different strategies, researchers have developed resistance-free genome editing techniques, which are commonly used in three ways:

1. Two-step homologous recombination based on FIp recombinase

Flp recombinase can recognize short Flippase recognition site sequences and excise the sequences between two flippase recognition targets (FRTS) [127]. Combined with Red homologous recombination system, the resistance genes with FRT sequences at both ends were integrated into the target site on the genome, and then the resistance genes were knocked out by Flp recombinase. The disadvantage of this technique is that it will leave a FRT sequence at the target site, which is not traceless editing and will affect the editing efficiency of genes after multiple rounds of editing.

(B) Two-step positive and negative screening method based on sacB gene

The sacB gene encodes secreted sucrose fructanase, which can catalyze the hydrolysis of sucrose into glucose and fructose, and synthesize fructose into high molecular weight fructan, which can produce lethal effect on *E. coli*, and is a negative screening marker. Under the guidance of Red homologous recombination system, the fragments containing resistance genes and sacB genes were first integrated into the target site [128], then the target fragments were integrated into the target site, and the gene fragments containing resistance genes and sacB genes were replaced, and then cultured in the medium containing sucrose. The correct transformants replacing the resistance gene and sacB gene fragment were obtained by reverse screening strategy.

(C) One-step homologous recombination based on CRISPR/Cas9

Genome editing technique, such as CRISPR–Cas, is extensively used in a diverse array of fields, with practical applications in the biological synthesis of new compounds. It is a powerful tool for studying eukaryotic microorganisms and has been reported to be useful for knocking out a specific targeted gene, simultaneously operating multiple genes, disrupting, activating, and repressing genes, and editing the genome in a genetically known or unknown microorganism [129]. Cas9 protein was cleaved at target sites under the mediation of gRNA, which caused the double chain break of *E. coli* genome and produced biofuels [130, 131]. The Red homologous recombination system can integrate the donor fragment into the target site, repair the broken DNA double strand, or undergo homologous recombination before Cas9 protein splicing, so that Cas9 loses the target site and does not splice.

The integration of synthetic biology, genome-editing technique like CRISPR/Cas9, and high-throughput sequencing technology, would lend feasibility to expanding carbon chain for the production of new advanced biofuels in industrial-scale applications. For example, some studies have been showed lignocellulosic biofuel production can be improved by CRISPR/Cas9-mediated lignin modification [132, 133]. Furthermore, it is highly possible that novel biosynthesis pathway could be discovered using this technique, which could lead to the acquisition of a wide variety of novel synthetic systems with high catalytic performance. This could in turn motivate novel gene expression system for carbon chain expansion to generate undiscovered advanced biofuels. Today, gene knockout and gene cassette insertions employing CRISPR–Cas9 in *Saccharomyces cerevisiae* and *Kluyveromyces marxianus* have resulted in enhanced production of bioethanol and 2-phenyl ethanol in these organisms, respectively [134]. Designing new engineering strategy and controlling synthesis processes could be greatly simplified, thus opening up an unprecedented field for the production of advanced biofuels.

One advantage of carbon chain expansion is that it allows refactoring of any potential catalytic reaction to be performed in a simple, convenient, inexpensive, efficient manner. This technology also lends practicality towards transformation of cellular structures of microorganisms and recombination of metabolic networks, especially in changing mitochondria to improve their synthesis efficiency. For example, substantial increase in production of advanced biofuels had been attributed to the close link between genetic improvement of the whole signaling network and recombination of the whole metabolic flux, such as genome editing of algal species by CRISPR–Cas9 for biofuels [135].

The potential application of this technology is continually being explored in different microorganism platforms, along with bioinformatics and large-scale modeling, for analysis at the genome level, transcriptome level, proteome level, metabolome depth, and fluxome-level. Findings from these studies would contribute to better understanding of the biochemical evolution and functional strain during gene function elucidation and epigenetic regulation in loss-of-function (LOF) [136] or gain-of-function (GOF) research [137]. This knowledge would also advance the carbon chain expansion process for producing advanced biofuels [189] and allow for the development of new target production from fungi via CRISPR–Cas9, instead of bacteria such as *Streptomyces* with fine-tuning the regulation [138–142], *Trichoderma reesei* with improving cellulase production [143], and *Aspergillus* species with improving succinic acid production from renewable biomass [144, 145] and yeast with genetic recombination for cellodextrin transport [190].

## Outlook

The rapid development of biosynthesizing advanced biofuels, coupled with the demand of newly reconstructed engineering pathway for synthesizing novel chemicals and the need to improve efficiency of existing strategies, require strengthening of previous biometabolic pathways and discovery of novel biosynthesis pathways. In this paper, the low efficiency problem of production in expanding carbon chain was presented, followed by the proposal of two biosynthesis strategies RM and ED, and a review of corresponding biosynthetic pathway for producing advanced biofuels. Our findings contribute to future design of novel catalytic routes to maximize the production of target product. In addition, genome editing technique such as the representative CRISPR/Cas9 gene-editing technology will generate a traction for effective resolution of many technical obstacles in discovering new engineering strategy and increasing production of advanced biofuels.

## Data Availability

No data are associated with this article.
